# The Balanced Scorecard as a Performance Management Tool in the Healthcare Sector – The Case of the Medical Commission Department at the Ministry of Public Health, Qatar

**DOI:** 10.7759/cureus.5262

**Published:** 2019-07-29

**Authors:** Salma K Al-Kaabi, Mohamad A Chehab, Nagah Selim

**Affiliations:** 1 Preventive Medicine, Ministry of Public Health, Doha, QAT; 2 Preventive Medicine, Hamad Medical Corporation, Doha, QAT; 3 Public Health and Preventive Medicine, Cairo University School of Medicine, Cairo, EGY

**Keywords:** performance measurement system, qatar, balanced scorecard, medical commission

## Abstract

Background

The balanced scorecard (BSC) system provides the basis for developing and executing a good strategy and successfully managing change at the institutional level. Moreover, developing a BSC performance system at the Medical Commission Department (MCD) in Qatar will enable stakeholders to approach their organization and work in a more strategic manner.

Methods

A descriptive cross-sectional survey was conducted to determine the need of employees at the MCD for the BSC, as a performance management tool, to attain the organizational objectives. Thus, a simple random sample was employed to enroll 199 participants. Also, a self-administered validated English and Arabic survey tool was employed to collect socio-demographic characteristics and encompassed 20 questions on the needs assessment for BSC.

Results

The response rate was high (97.5%) while the scoring indicates difficulty in successfully executing the MCD strategy and meeting the needs of their customers. The results showed a medium score of need to implement the BSC (score =59.9±9.7). The BSC needs score was strongly associated with the item “Our employees have a solid understanding of the mission, vision, and strategy” and it was higher among nationals as well as those with a higher level of education.

Conclusion

The BSC system provides the basis for properly executing a strategy and successfully managing change in an organization. Thus, building a BSC performance system at the MCD will enable employees to think in a more strategic way about their organization and their work. It will also bring change in the way things are done, as new policies and procedures will be developed and be implemented accordingly.

## Introduction

Management sciences have progressed greatly in the past few decades. In the 20th century, many great management theories have proven highly effective in overall organizational management. One of these theories is the Balanced Scorecard (BSC) approach, which has greatly evolved since its inception in the early nineties by Kaplan and Norton [1]. The aforementioned approach has delivered substantial efficiency and effectiveness due to its focus on future targets or long-term performance based on the alignment of current processes with a balanced strategy and performance indicators. Hence, the idea is to improve present practices along with a mechanism of check and balance which keeps the current performance aligned to the objectives [2].

The BSC was originally developed as a framework to measure the financial as well as the non-financial performance of private industries. Moreover, the framework is equally applicable to public sector organizations but necessitates certain modifications to account for the lack of profitability as an objective in the government and public sector entities. Similarly, such organizations focus on the value of the service provided to the customer rather than profit or return on investment for the shareholder. Globally, many institutions and organizations have adopted this approach for business management. Out of these organizations, healthcare settings are one of the most distinguished setups. The benefits and the extensive approach that it can offer not only have a potential to alter the strategic operations of single healthcare organizations, but it may also be helpful in managing the entire healthcare system as well.

The BSC framework portrays a three-dimensional view of a business’s organizational performance: results (financial and customer), operations, and capacity. In the balanced scorecard terminology, vision, mission, and strategy at the corporate level are decomposed into different views or perspectives as seen through the eyes of business owners, customers, as well as other stakeholders, managers, process owners, and employees [3].

Despite the absence of performance management systems specific to healthcare organizations, many of the latter have evolved as learning organizations; basically to enhance the performance and quality of organizational services [4]. However, the performance management system existing in today’s healthcare organizations comprises of the simplest forms. This includes a concrete and defined mission along with effective communications to employees who are stakeholders in the organization and are aware of business objectives and other strategic plans. On the other hand, the BSC approach offers a framework for strategy implementation as well as a tool for measuring the success of the strategy’s implementation [5]. Similarly, the BSC represents a management model which can be used to translate an organization's mission and strategy into a comprehensive set of performance measures that provide a basis for a strategic measurement and management system [1].

As the healthcare industry affects the welfare of people worldwide, it is vital that the former functions optimally. In Qatar, the health care sector is facing different types of challenges and receiving great attention from the national authorities. Thus, the country’s healthcare sector is undergoing continuous modification. In addition to that, the health industry in Qatar is mainly governmental, nonprofit, and associated with significant government expenditures; hence constituting an economic burden on the state budget. Subsequently, the aforementioned situation made any procedure pertaining to the management of the healthcare system a critical process, since it impacts the progress towards achieving national strategic objectives. Subsequently, one of the most important issues that have emerged is to maintain continuous improvement of the healthcare system based on the proper evaluation and management of its performance.

In 2011, Qatar launched the National Health Care Strategy (NHS) (2011-2016), which is intended to propel Qatar toward the health goals and objectives underlined in Qatar’s National Vision (QNV) 2030. The strategy incorporates the subsequent principles which underpin the Ministry of Public Health’s National Health Vision 2020: Caring for the Future - Establishing a Healthy Vibrant Society, and aims to enhance the wellness of the people in Qatar so that a vibrant, healthy, and productive society can be established today and in the future. Thus, the purpose of conducting this research is to assess the need for a balanced scorecard approach as a tool of performance management at the Medical Commission Department (MCD), with the ultimate goal of applying it as a role model for other departments at the Ministry of Public Health (MoPH) as well as the various health care organizations in the State of Qatar.

## Materials and methods

Study design

The current study is a descriptive cross-sectional study and was conducted at the MCD in order to assess the need for a balanced scorecard approach. A BSC needs assessment questionnaire was developed and circulated among all employees. The collected data were analyzed and the results aided in adopting a BSC approach at the aforementioned department.

 Study setting

The MCD operates under the umbrella of the Ministry of Public Health and is one of the major departments in the Medical Affairs Directorate. Moreover, it has a vital role in the control and prevention of communicable diseases in Qatar. The department’s mission is to protect the health of the population and prevent the spread of infectious diseases from those newly arriving to the country. Thus, all expatriates working and living in the country are legally required to undergo a health screening process upon their arrival to the country as a prerequisite to receiving the residency permit. Subsequently, the newcomers should first obtain a blood-type certificate from any clinic prior to undertaking the aforementioned screening exam, which is comprised of a blood test and a chest X-ray. Furthermore, the blood-borne diseases being screened include human immunodeficiency virus (HIV)/acquired immune deficiency syndrome (AIDS), tuberculosis, hepatitis B, and hepatitis C. In addition to that, expatriates who are employed at restaurants, barbershops, laundries, health clubs, and other public places are further required to undergo annual health checks. Moreover, it is the sole responsibility of the MCD to perform medical examinations and issue medical certificates for the following groups:

· Prospective and current employees

· Sick and injured employees, including signing sick leave forms

· Foreigners coming to work and reside in the country

· Applicants for Qatari citizenship

· Driving license applicants

· Students prior to attending or joining university or obtaining a scholarship

· Any other cohort who is legally required to receive a physical examination, excluding those employed at the Ministries of Interior and Defense

Study sample

The study targeted MCD employees (doctors, nurses, laboratory and radiology consultants, technicians, and administrators). The department has a total sample of 410 employees. Assuming the prevalence of BSC need in the study population is 50%, a sample of 199 subjects was needed such that precision was 5% and alpha=0.05 [6]. The study subjects were selected randomly using a simple random sampling method. In addition to that, the selected employees were enrolled regardless of nationality, educational level, and employment degree. A random generating program was used to select participants from an input list of MCD employees. After which, the study subjects were identified and the questionnaire was distributed upon eliciting their verbal consent to participate in this study. 

Data collection tools 

The researcher adopted a structured, standardized, and self-administered questionnaire available in the Arabic and English languages. It encompassed two sections; the first assessed the demographic characteristics of the respondents such as age, gender, educational level, nationality, marital status, and the section where the participant works. The second section of the survey was based on previous literature and included 20 questions to assess the degree of need for the BSC as a performance management tool [7]. Each item of the questionnaire was scored using the 1-5 Likert scale (strongly disagree-disagree-neutral-agree-strongly agree), where the higher the level of the agreement the higher the score that will be assigned. For example, if the respondent strongly agrees, a score of 5 points will be given. The questionnaire’s face validity was attained through piloting it on a convenience sample for its content and length. As a result, some questions were modified and the questionnaire format was finalized. The questionnaire was distributed to the study subjects who were allotted 10-15 minutes to be complete the latter.

Statistical analysis

The collected data was coded and entered and processed using the Statistical Packages for Social Science [SPSS v. 19.0]. Then, descriptive statistics were presented with means and standard deviations for the continuous variables while categorical data were summarized through proportions and percentages. The t-test was employed to compare the BSC’s need score among two variables such as gender and nationality. Furthermore, a one-way ANOVA was conducted to compare the means between participants based on the education status and the response to items in the questionnaire pertaining to their understanding of the institution’s mission, vision, and strategy. Moreover, the Bonferroni post hoc tests were performed if the main effects were found to be significant. Finally, Spearman’s correlation was utilized to evaluate the strength of the association between the BSC needs score and age. The level of significance was set at p<0.05.

## Results

**Figure 1:**
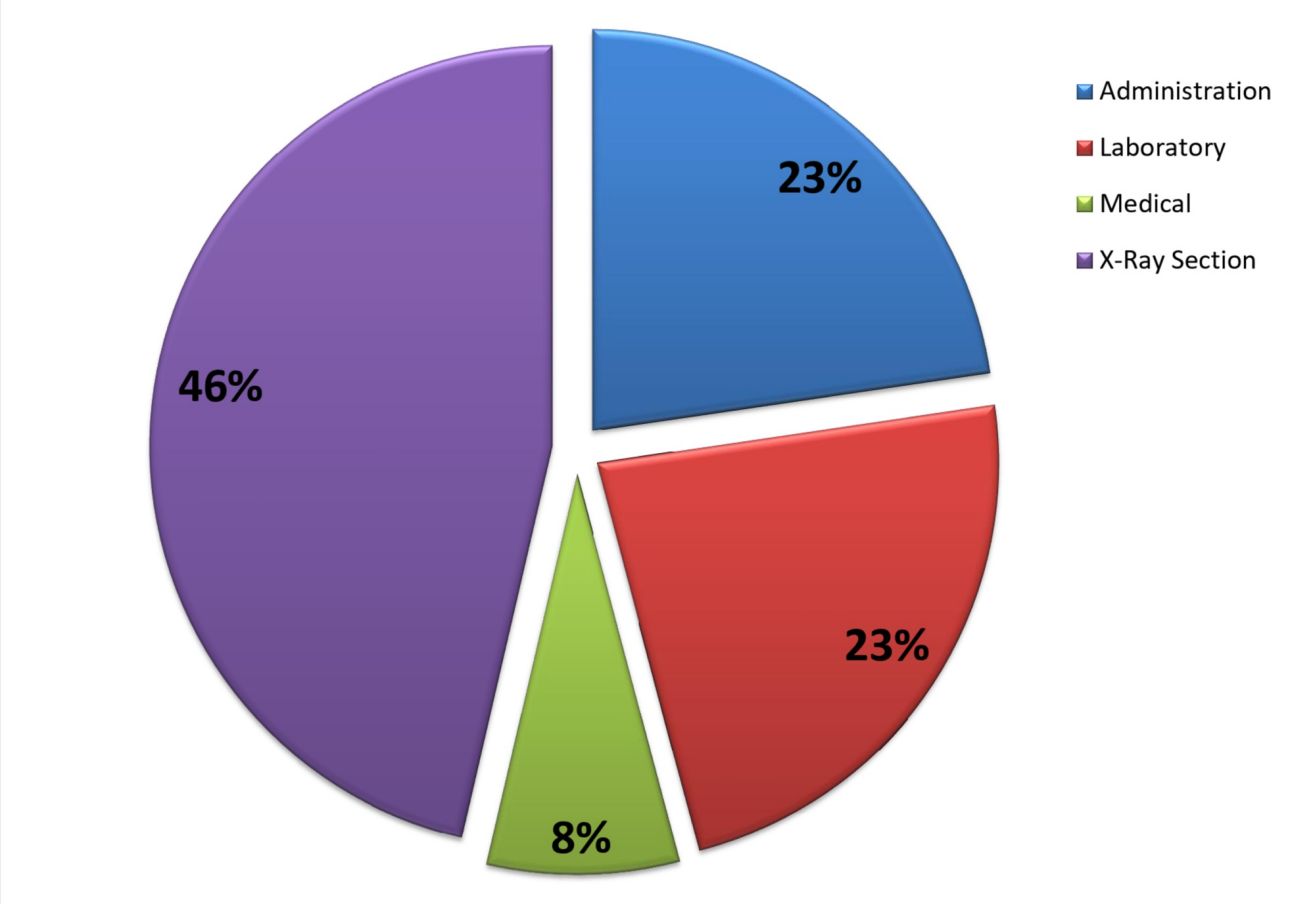
Distribution (%) of the study population (N=194) by staff category

In total, 194 employees completed the distributed questionnaires, with a response rate of 97.5%. The socio-demographic characteristics of the participants are shown in Table 1. The mean age of the study population was 34.4 years and there was an equal distribution (50%) of both genders. On the other hand, the majority of the participants were non-Qatari (69.6%). In addition to that, more than half of the respondents (55.2%) reported a university-graduate level of education while almost one-third (34%) were found to have intermediate education. The categorical job description of the interviewed staff is shown in Figure 1.

**Table 1:** The socio-demographic characteristics of the study population (N=194)

Variable		Count	Percentage (%)
Gender	Male	97	50.0%
	Female	97	50.0%
Nationality	Qatari	59	30.4%
	Non-Qatari	135	69.6%
Education	Illiterate	2	1.0%
	Elementary	7	3.6%
	Preparatory	7	3.6%
	Secondary	52	26.8%
	University	107	55.2%
	Postgraduate	19	9.8%

The responses to the second section of the questionnaire are shown in Table 2. Regarding the need for a balanced scorecard assessment, it was highlighted through the reply of the study subjects to the following two items: “We have numerous initiatives taking place at our organization, and it’s possible that not all are truly strategic in nature” and “We cannot clearly articulate our strategy in a one-page document or “map””. Regarding the first item, less than half (43.3%) of the participants reported disagreeing, while one-quarter (25.3%) agreed. In regards to the second item, more than a quarter of the employees (25.8%) agreed on not being able to portray their institution’s strategy on paper while a similar percentage (21.6%) was neutral.

**Table 2:** Frequency distribution of responses to 20 items from the BSC needs’ assessment questionnaire (N=194)

Question	Strongly Disagree	Disagree	Neutral	Agree	Strongly Agree
Our organization has invested in Total Quality Management (TQM) and other improvement initiatives but we have not seen a corresponding increase in financial or customer results.	48(24.7)	59(30.4)	47(24.2)	26(13.4)	14(7.2)
--- If we did not produce our current Performance Reports for a month, nobody would notice.	34(17.5)	55(28.4)	44(22.7)	41(21.1)	20(10.3)
We create significant value from intangible assets such as employee knowledge and innovation, customer relationships, and a strong culture.	20(10.3)	51(26.3)	59(30.4)	42(21.6)	22(11.3)
-- We have a strategy (or have had strategies in the past) but have a hard time successfully implementing it.	40(20.6)	39(20.1)	56(28.9)	38(19.6)	21(10.8)
-- We rarely review our performance measures and make suggestions for new and innovative indicators.	37(19.1)	49(25.3)	48(24.7)	43(22.2)	17(8.8)
Our senior management team spends the majority of their time together discussing variances from plan and other operational issues.	20(10.3)	36(18.6)	76(39.2)	37(19.1)	25(12.9)
-- Budgeting at our organization is political and based largely on historical trends.	34(17.5)	42(21.6)	62(32.0)	40(20.6)	16(8.2)
--- Our employees do not have a solid understanding of our mission, vision, and strategy.	22(11.3)	32(16.5)	62(32.0)	51(26.3)	27(13.9)
-- Our employees do not know how their day-to-day actions contribute to the organization’s success.	25(12.9)	31(16.0)	25(12.9)	77(39.7)	36(18.6)
-- Nobody owns the performance measurement process at our organization.	22(11.3)	32(16.5)	45(23.2)	66(34.0)	29(14.9)
-- We have numerous initiatives taking place at our organization, and it’s possible that not all are truly strategic in nature.	37(19.1)	47(24.2)	61(31.4)	37(19.1)	12(6.2)
-- There is little accountability in our organization for the things we agree as a group to do.	27(13.9)	56(28.9)	36(18.6)	58(29.9)	17(8.8)
-- People tend to stay within their “silos,” and as a result we have little collaboration among departments.	26(13.4)	31(16.0)	44(22.7)	62(32.0)	31(16.0)
-- Our employees have difficulty accessing the critical information they need to serve customers.	25(12.9)	37(19.1)	41(21.1)	55(28.4)	36(18.6)
Priorities at our organization are often dictated by current necessity or “fire-fighting.”	45(23.2)	60(30.9)	41(21.1)	30(15.5)	18(9.3)
The environment in which we operate is changing, and in order to succeed, we too must change.	46(23.7)	60(30.9)	29(14.9)	36(18.6)	23(11.9)
--We face increased pressure from stakeholders to demonstrate results.	40(20.6)	41(21.1)	55(28.4)	44(22.7)	14(7.2)
-- We do not have clearly defined performance targets for both financial and nonfinancial indicators.	36(18.6)	37(19.1)	59(30.4)	38(19.6)	24(12.4)
-- We cannot clearly articulate our strategy in a one-page document or “map”.	47(24.2)	55(28.4)	42(21.6)	37(19.1)	13(6.7)
-- We sometimes make decisions that are beneficial in the short term but may harm long-term value creation.	39(20.1)	44(22.7)	56(28.9)	39(20.1)	16(8.2)

**Figure 2:**
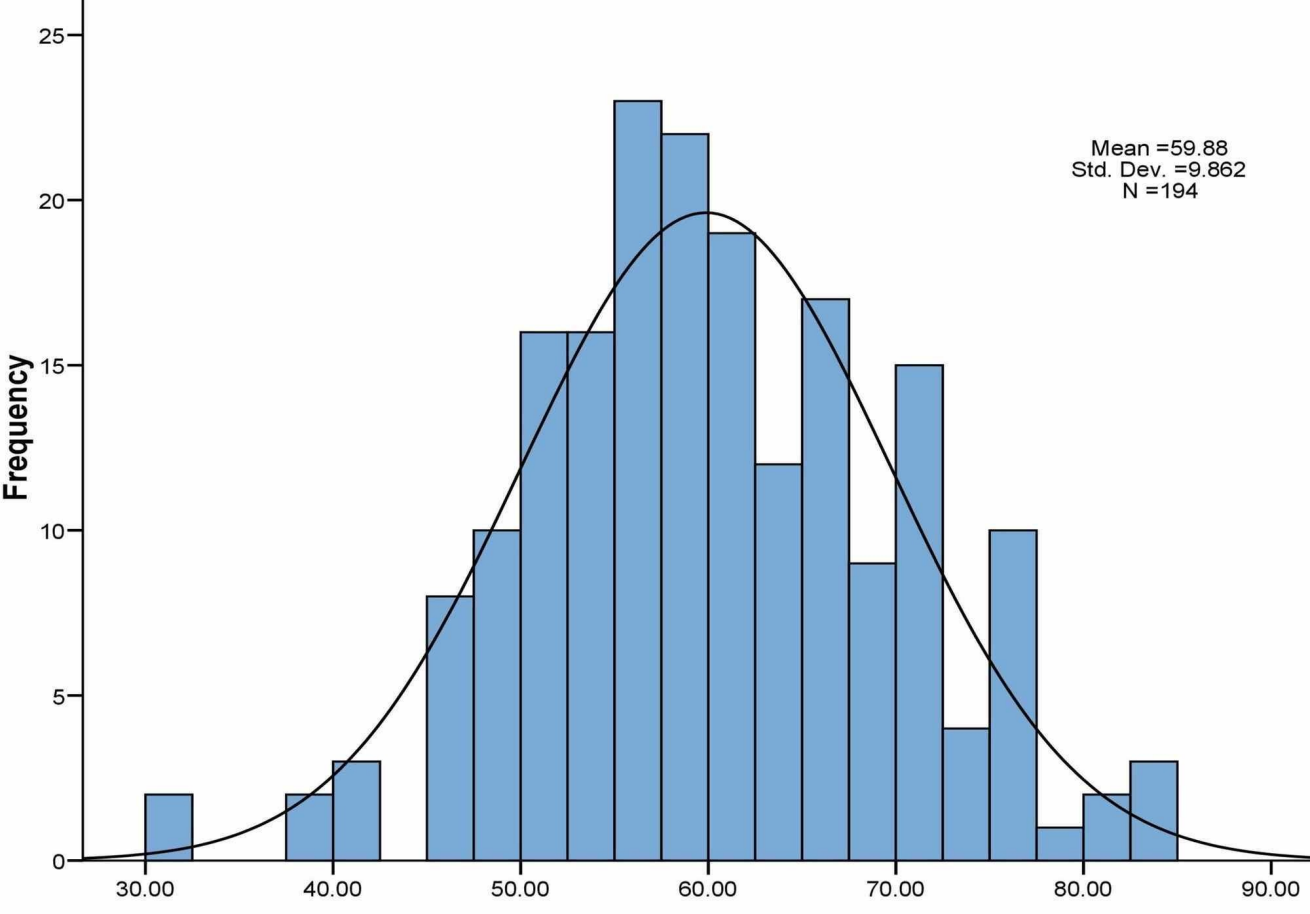
Histogram showing the distribution of the BSC Need Score among the study population

The BSC needs score among the studied population was normally distributed with mean of 59.9±9.7 SD (Figure 2). Furthermore, according to the BSC needs assessment classification criteria, [7] it was found more than half (about 57.2%) of the surveyed employees scored in the medium category while the rest (42.8%) scored in the high. By gender, the aforementioned needs score was similar among males (59.1±8.7) and females (60.7±10.9) but not statistically significant (p=0.266). Moreover, the BSC needs score was negatively correlated with age (r=-0.13, p=0.076) but did not reach statistical significance. On the other hand, Qatari nationals (63.6±10.5) scored higher when compared to their non-Qatari counterparts (58.3±9.1) on the needs assessment, given the fact that more nationals occupied administrative jobs. Regarding the association between the BSC needs score and the education level, a statistically significant relationship was revealed in which the needs score was higher among those with a secondary, university, or postgraduate education status when compared to those with a lower education level (less than secondary school) (p<0.009).

## Discussion

In the current study, the aim was to assess the need for a BSC approach as a performance management tool at the MCD and this was done through assessing the employee’s response regarding the BSC strategic areas. Thus, the scoring indicates a difficulty in successfully executing the department’s strategy and meeting the needs of their customers because the results showed a medium (nearly high) score of need to implement the BSC (score =59.9±9.7). As a result, this signifies that the organization may have a performance measurement system in place but is not experiencing the benefits anticipated or the need to succeed [7].

In addition, the BSC needs score was shown to be strongly associated with the employees’ understanding of the mission, vision, and strategy at the MCD; which strongly necessitates the implementation of the balanced scorecard approach. Various organizations (profit-oriented and non-profit) across the globe are opting for BSC application in their operation with the healthcare sector not being that far behind. Indeed, there is a great need for the BSC approach at the ministries of health so that the benefits of this effective management tool can be utilized at a greater extent. The Ministry of Public Health is one of the most sophisticated areas of any national management business in Qatar. In many developed countries, the expenditures related to the public health sector are higher than those allocated; which always results in budget deficits. Therefore, it is necessary to properly utilize the allocated resources for the sustainability of the national health system.

The author will share the results of this study with the higher authority at the Ministry of Public Health and the MCD. The management board should bear in mind that in order to implement the balanced scorecard approach, they must be ready to provide the leadership, commit the resources, and train their staff on the implementation of the BSC system to boost their contribution in the organization. Furthermore, many lessons can be learned from earlier experiences with the BSC approach in the healthcare setting in other Arabian, Gulf, or Western countries, especially with respect to the identification, measurement, and reporting of performance indicators. Definitively, the management should attempt to strike a better balance between the use of financial and non-financial indicators as well as qualitative and quantitative indicators when developing the BSC in their respective organizations. Also, the management board should know that the decision to develop a BSC system is a continuous one. While there are discreet start and stop points along the way, the real value of a scorecard system comes from the continuous self-inquiry and in-depth analysis that is at the core of all successful strategic planning and performance management systems.

Regarding the staff at the MCD, their involvement in the BSC approach is a lengthy but critical process that is at least as important as measuring performance. It is also vital to center such approach on the customers' needs because they drive the way an organization responds with products and services; thus, the vision, mission, and values of an organization will shape its culture and lead to a set of strategic goals that outline expected performance. In addition to that, the business strategies will provide employees with the chosen approach to meet customers’ needs and attain the desired goals. Moreover, the aforementioned strategies will constitute building blocks that can be mapped and measured through specific indicators that will reveal the desired levels of performance. 

## Conclusions

The BSC system provides the basis for properly executing a strategy and successfully managing change in an organization. Thus, building a BSC performance system at the MCD will enable employees to think in a more strategic way about their organization and their work. It will also bring change in the way things are done, as new policies and procedures will be developed and be implemented accordingly.
